# A Cytoplasmic Form of *Gaussia* luciferase Provides a Highly Sensitive Test for Cytotoxicity

**DOI:** 10.1371/journal.pone.0156202

**Published:** 2016-05-26

**Authors:** Saori Tsuji, Tetsuya Ohbayashi, Kohji Yamakage, Mitsuo Oshimura, Masako Tada

**Affiliations:** 1 Bio Frontier Project Promotion Section, Organization for Tottori Industrial Promotion, Nishi-cho 86, Yonago, Tottori, 683–8503, Japan; 2 Division of Laboratory Animal Science, Research Center for Bioscience and Technology, Tottori University, Nishi-cho 86, Yonago, Tottori, 683–8503, Japan; 3 Hatano Research Institute, Food and Drug Safety Center (FDSC), 729–5 Ochiai, Hatano, Kanagawa, 257–8523, Japan; 4 Chromosome Engineering Research Center, Tottori University, Nishi-cho 86, Yonago, Tottori 683–8503, Japan; Robert Koch-Institute, GERMANY

## Abstract

The elimination of unfavorable chemicals from our environment and commercial products requires a sensitive and high-throughput *in vitro* assay system for drug-induced hepatotoxicity. Some previous methods for evaluating hepatotoxicity measure the amounts of cytoplasmic enzymes secreted from damaged cells into the peripheral blood or culture medium. However, most of these enzymes are proteolytically digested in the extracellular milieu, dramatically reducing the sensitivity and reliability of such assays. Other methods measure the decrease in cell viability following exposure to a compound, but such endpoint assays are often confounded by proliferation of surviving cells that replace dead or damaged cells. In this study, with the goal of preventing false-negative diagnoses, we developed a sensitive luminometric cytotoxicity test using a stable form of luciferase. Specifically, we converted *Gaussia* luciferase (G-Luc) from an actively secreted form to a cytoplasmic form by adding an ER-retention signal composed of the four amino acids KDEL. The bioluminescent signal was >30-fold higher in transgenic HepG2 human hepatoblastoma cells expressing G-Luc+KDEL than in cells expressing wild-type G-Luc. Moreover, G-Luc+KDEL secreted from damaged cells was stable in culture medium after 24 hr at 37°C. We evaluated the accuracy of our cytotoxicity test by subjecting identical samples obtained from chemically treated transgenic HepG2 cells to the G-Luc+KDEL assay and luminometric analyses based on secretion of endogenous adenylate kinase or cellular ATP level. Time-dependent accumulation of G-Luc+KDEL in the medium increased the sensitivity of our assay above those of existing tests. Our findings demonstrate that strong and stable luminescence of G-Luc+KDEL in human hepatocyte-like cells, which have high levels of metabolic activity, make it suitable for use in a high-throughput screening system for monitoring time-dependent cytotoxicity in a limited number of cells.

## Introduction

Newly synthesized and naturally occurring chemicals play important roles in industry, including in pharmaceutical development. Before they can be widely used, the safety of such compounds must be carefully assessed. Currently, to obtain information regarding the health hazard of specific chemicals, the Repeated Dose 28-day Oral Toxicity Study [Organization for Economic Cooperation and Development (OECD) Test Guideline 407] is often performed, primarily using rodents [1]. However, it is desirable to decrease our reliance on animal testing for several reasons, including time, space, cost, the low throughput of these methods, the uncertain relevance of animal-based results to humans, and increasing societal objection to the use of animals in research. One solution to these problems would be to use human primary somatic cells to investigate the cytotoxic potential of multiple chemicals. The OECD has also promoted the establishment of a universal chemical risk assessment framework using non-animal systems [2, 3]. However, because not only a given compound but also its metabolites can cause cytotoxicity or transcriptional induction and/or inhibition of P450 enzymes, implementation of these assays requires large-scale culture of primary human hepatocytes [4]. Unfortunately, in this regard, human primary hepatocytes are unstable in culture and cannot easily proliferate. Therefore, we sought to establish a practical cytotoxicity assay system that is highly sensitive, inexpensive, easy to use, and applicable to quantitative high-content screening (HCS) using a conventional human hepatic cell line. However, because human hepatic cell lines often possess low hepatic function, an accurate test can be realized only when the system is applied to human hepatic cells with relatively high metabolic activity, such as HepaRG [5, 6]. Prior to establishing an optimized system, we evaluated our new reporter construct in transgenic HepG2 cells, a hepatoblastoma cell line. These cells, which are widely used to detect cytotoxic chemicals in humans, are more suitable models of human primary hepatocytes than other metabolically incompetent cells [7].

Hepatotoxicity is often evaluated by calculating the ratio of two serum biochemical factors, alanine aminotransferase (ALT) and aspartate aminotransferase (AST), which are secreted from damaged cells into peripheral blood [8]. ALT is predominantly, but not exclusively, expressed in the liver. Although similar principles underlie several *in vitro* cell-based cytotoxicity tests that measure cytoplasmic enzymes, including lactate dehydrogenase (LDH) and adenylate kinase (AK), these secreted proteins are actively digested by proteolytic enzymes in the culture medium, leading to loss of their activities over time [9–11]. Alternatively, cellular ATP can be measured as a marker of cell viability at the endpoint of a cytotoxicity assay [12]. However, such endpoint assays may lead to false-negative diagnoses because they do not take into account the replacement of dead or damaged cells by new cells produced by the proliferation of surviving cells. To increase the sensitivity of cytotoxicity tests, it is necessary to improve the stability of the reporter proteins used for this purpose. In this study, we used a genetically modified cytoplasmic luciferase to develop a one-step highly sensitive HCS-based test for hepatotoxicity of candidate compounds.

*Gaussia* luciferase (G-Luc) and *Cypridina* luciferase (C-Luc), derived from the marine copepod *Gaussia princeps* and the ostracod *Cypridina noctiluca*, respectively, are stable in blood, urine, and cell extracts [13–16]. Moreover, the bioluminescent signal of humanized G-Luc expressed in mammalian cells is 1,000-fold higher than those of other widely used cytoplasmic luciferases, e.g., *Renilla* and firefly luciferases (R-Luc and F-Luc) [14]. However, because G-Luc and C-Luc contain a native signal peptide (SP) at their N-termini, they are actively secreted from expressing cells under physiological conditions, even in the absence of cellular damage [14, 17]. Previously, a cytoplasmic variant of G-Luc was generated by addition of the endoplasmic reticulum (ER)-binding signal KDEL to the C-terminus, yielding G+KDEL [14]. By contrast, elimination of the SP element (∆SP) from the N-terminus of G-Luc, an alternative strategy for localizing the enzyme to the cytosol, reduced its enzymatic activity [14]. We predicted that hepatocyte-like cells expressing cytoplasmic variants of G-Luc and/or C-Luc could serve as the basis for an accurate, one-step HCS-based cytotoxicity test.

**Fig 1:**
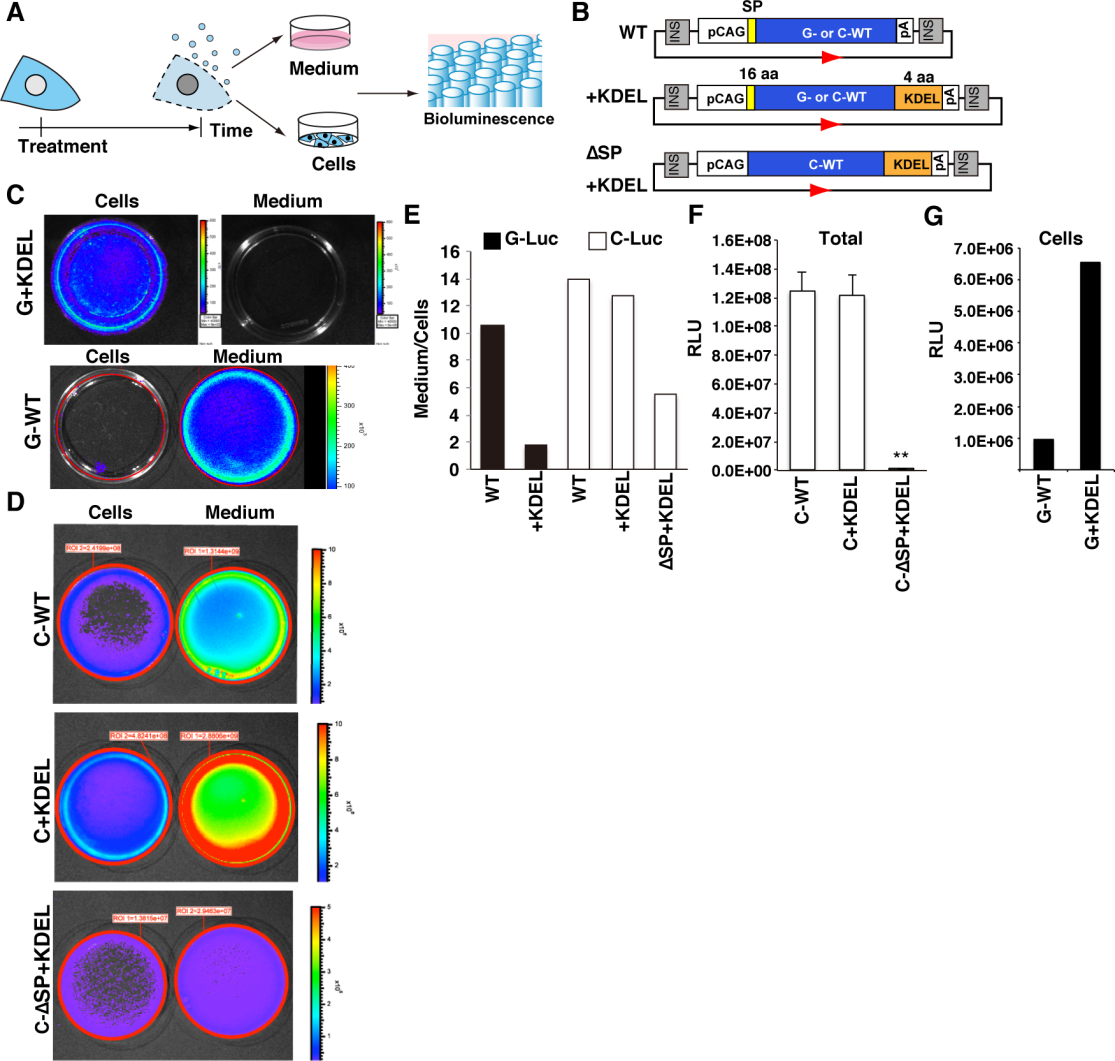
Five luciferase expression vectors and the properties of G-Luc, C-Luc, and their variants in HepG2 cells. (A) Experimental scheme of the cytotoxicity test presented in this study. (B) Luciferase expression vectors prepared in this study, initially characterized by transient expression in HepG2 cells. Blue: ORFs of G-WT or C-WT; orange: KDEL-encoding sequences listed in Table 1; yellow: SP, native signal peptide-encoding sequences; INS, insulator sequence; CAG, CAG promoter isolated from vector pCAGGS. (C) IVIS luminescence imaging of HepG2 cells expressing G+KDEL or WT-G. The blue signal indicates intracellular G-Luc activity. The absence of a signal in the “Medium” sample of G+KDEL-expressing cells indicates that the activity of secreted G-Luc in the culture medium was below the measurable limit. (D) IVIS luminescence imaging of HepG2 cells expressing C-Luc variants. Medium had higher C-Luc activity (red or yellow) than cells (blue). (E) Ratio of luminometric activities in medium *vs*. cells, obtained using a microplate reader after 48 hr culture. The reduction in the Medium/Cells ratio reflects the conversion of G-Luc and/or C-Luc from the naturally secreted form to a cytoplasmic form. (F) Extinction of luminometric activity in HepG2 cells expressing C-∆SP+KDEL, detected using a microplate reader (n = 4 wells for each, **p < 0.001). RLU: relative light units. (G) Strong intracellular luminometric activity in HepG2 cells expressing G+KDEL, detected using a microplate reader.

To test this idea, we created expression vectors for the luciferase variants G+KDEL and C+KDEL and analyzed their characteristics in HepG2 cells (Fig 1A). G+KDEL exhibited strong intracellular luminescence in transgenic HepG2 cells, and its enzymatic activity was stably maintained in crude cell extracts for at least 24 hr at 37°C. By contrast, the C+KDEL variant maintained its native secretory properties even after the SP element was deleted (C-∆SP+KDEL). The G+KDEL exhibited extraordinary stability and strong blue luminescence, as also observed for wild-type (WT) G-Luc. In addition, the time-dependent accumulation of secreted G+KDEL from damaged cells into the culture medium increased the sensitivity of the cytotoxicity assay above that of commercially available tests based on secreted AK or cellular ATP level. Thus, the cytoplasmic G+KDEL reporter is suitable for use in HCS-based hepatotoxicity tests in multi-well plates using a limited number of human hepatocyte-like cells.

## Results

### Cytoplasmic localization of G+KDEL

First, we constructed five expression vectors (Fig 1B). The open reading frames (ORFs) of WT G-Luc (G-WT) or WT C-Luc (C-WT) were obtained from the appropriate original vectors and subcloned into the pCAG shuttle vector for expression under the control of the CAG fusion promoter, a strong promoter created by fusion of the cytomegalovirus (CMV) enhancer with the promoter of chicken ACTINß [18]. The expression units were placed between insulator sequences (INS) to prevent inappropriate inactivation by position effects. A loxP site was also placed into the vector, facilitating easy integration of a single copy vector into an acceptor loxP site previously placed in a specific chromosomal region of the host cell genome by Cre-mediated site-specific recombination [19]. The ORFs of G-Luc and C-Luc with KDEL were amplified using 3’ reverse primers containing the 12-base sequence encoding KDEL (Table 1). The ORF of the C-∆SP+KDEL variant was amplified using a 5’ forward primer lacking a SP sequence and a reverse primer containing the KDEL-encoding sequence. The variant ORFs were also placed under the control of the CAG promoter (Fig 1B).

**Table 1:** Primer sets used in this study.

reporter genes	Primers	direction	sequences*
G-Luc+KDEL	G-Luc-F (*Nhe*I)	Forward	CCGC*GCTAGC*ATGGGAGTCAAAGTTCTGTTTG
	G-Luc+KDEL-R	Reverse	CGC*ttaGAGCTCGTCCTT*GTCACCACCGGCCC
C-Luc (WT)	C-Luc (*Bam*HI-*Bsr*GI)	Forward	CC*GGATCCTGTACAGCCACC*ATGAAGACCTTAATTCTTGC
	C-Luc (*Mlu*I-*Mfe*I)	Reverse	CCC*ACGCGTCAATTGcta*TTTGCATTCATCTGGTACTTCTA
C-Luc+KDEL	C-Luc (*Bam*HI-*Bsr*GI)	Forward	CC*GGATCCTGTACAGCCACC*ATGAAGACCTTAATTCTTGC
	C-Luc+KDEL(MluMfeI)	Reverse	CCC*ACGCGTCAATTGctaGAGCTCGTCCTT*TTTGCATTCATCTGGTACTTC
C-LucΔSP+KDEL	C luc Δ18 Kozak (*Bam*HI-*Bsr*GI)	Forward	CC*GGATCCTGTACAGCCACC*ATGCAGGACTGTCCTTACGAACC
	C-Luc+KDEL (*Mlu*I-*Mfe*I)	Reverse	CCC*ACGCGTCAATTGctaGAGCTCGTCCTT*TTTGCATTCATCTGGTACTTC
Element	Sequences		
*Nhe*I	GCTAGC		
*Bam*HI	GGATCC		
*Bsr*GI	TGTACA		
*Mlu*I	ACGCGT		
*Mfe*I	CAATTG		
KDEL	AAGGACGAGCTC		
stop codon	taa, tag		
Kozak	GCCACC		

*italic: elements listed above.

To characterize the G-Luc and C-Luc variants, each expression vector was transiently expressed in HepG2 cells, and the bioluminescence signals of luciferases secreted into the culture medium (Medium) were compared with those of intracellular luciferases (Cells). The Medium/Cells ratio represents the tendency of the luciferase variant to be secreted. Cytoplasmic localization of G+KDEL luminescence was visualized using an IVIS *in vivo* live imaging system (Fig 1C): cells expressing G+KDEL exhibited strong luminescence in the culture medium after addition of coelenterazine, a G-Luc-specific substrate, whereas medium placed in a new dish did not exhibit any luminescence. By contrast, the medium of cells expressing G-WT, C-WT, C+KDEL, or C-∆SP+KDEL exhibited stronger luminescence than the cells, indicating that these variants were secreted (Fig 1C and 1D). These observations were confirmed by additional analyses using a microplate reader (Fig 1E and 1F): the Medium/Cells ratio of G+KDEL was 16% of that of G-WT, whereas the Medium/Cells ratios of C-KDEL and C-∆SP+KDEL were 91% and 39% of that of C-WT, respectively (Fig 1E, n = 4 wells). However, although total luminescence did not differ significantly between C-WT and C+KDEL, C-∆SP+KDEL exhibited very little luminescence in both cells and medium (Fig 1F, n = 4 wells, *p* < 0.001), as determined by IVIS (Fig 1D). In addition, G+KDEL exhibited stronger intracellular luminescence than G-WT (Fig 1G). Therefore, of the five luciferase variants analyzed, G+KDEL was selected for subsequent analyses.

### G+KDEL is highly stable

**Fig 2:**
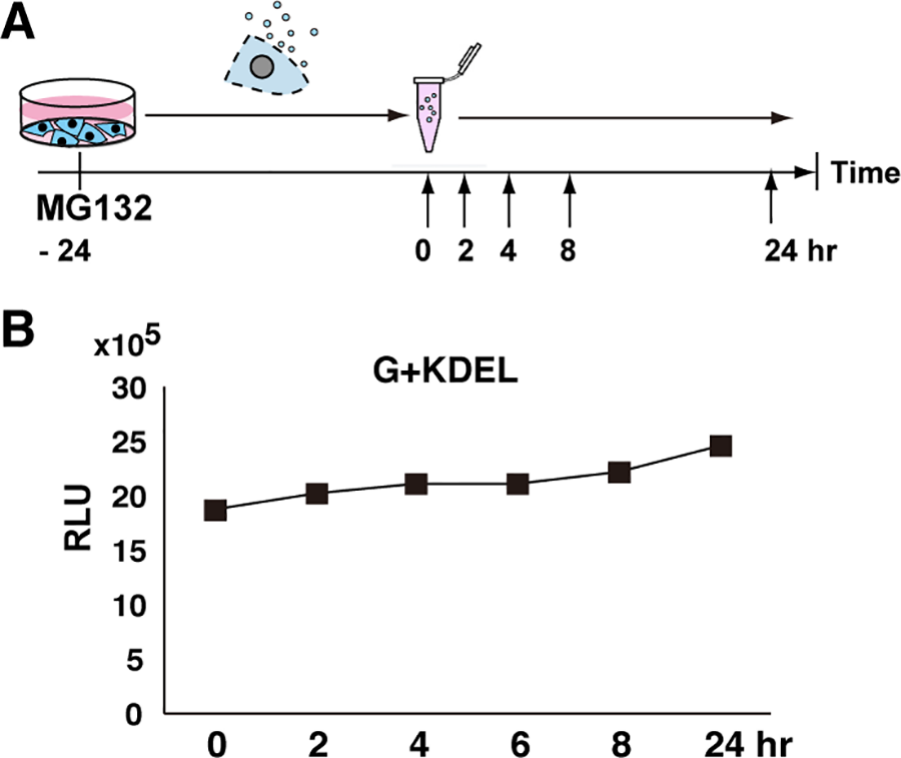
G-Luc+KDEL is highly stable in culture medium of damaged HepG2 cells. Medium was collected at five time points 24 hr after the treatment of G+KDEL-expressing HepG2 cells with a high concentration (0.5 μM) of the proteasome inhibitor MG132, and G-Luc activity was measured on a microplate reader.

Next, we evaluated the stability of secreted G+KDEL protein in the medium. HepG2 cells expressing G+KDEL were treated with 0.5 μM MG132, a cytotoxic proteasome inhibitor, which prevents the degradation of ubiquitinated proteins and induces apoptosis [20]. After 24 hr, the luminescence of cells treated with MG132 was 30% of that in non-treated controls (n = 3, triplicated measurements, data not shown). Next, we collected medium and incubated aliquots for up to 24 hr at 37°C (Fig 2A). Luminometric analyses of the samples revealed that G+KDEL was stable in medium for 24 hr at 37°C (n = 3, triplicate measurements), even while continuously exposed to proteolytic activities co-secreted from damaged cells (Fig 2B).

### Strong luminescence of G+KDEL in the transgenic HepG2 cells

Stable transgenic cells harboring a single copy of the G+KDEL or G-WT expression vector were created by Cre-mediated recombination and selection of G418-resistant clones. For this purpose, we used HepG2 cells in which a loxP site was inserted on chromosome 14 [19]. Consequently, in contrast to clones generated by random insertion, the expression properties of the transgenic clones should be directly comparable. Thirteen and nine clones were isolated for G+KDEL and G-WT, respectively; of these, clone 12 for G+KDEL (G+KDEL12) and clone 4 for G-WT (G-WT4) were selected for further analysis on the basis of genomic PCR and bioluminescence signal intensities.

**Fig 3:**
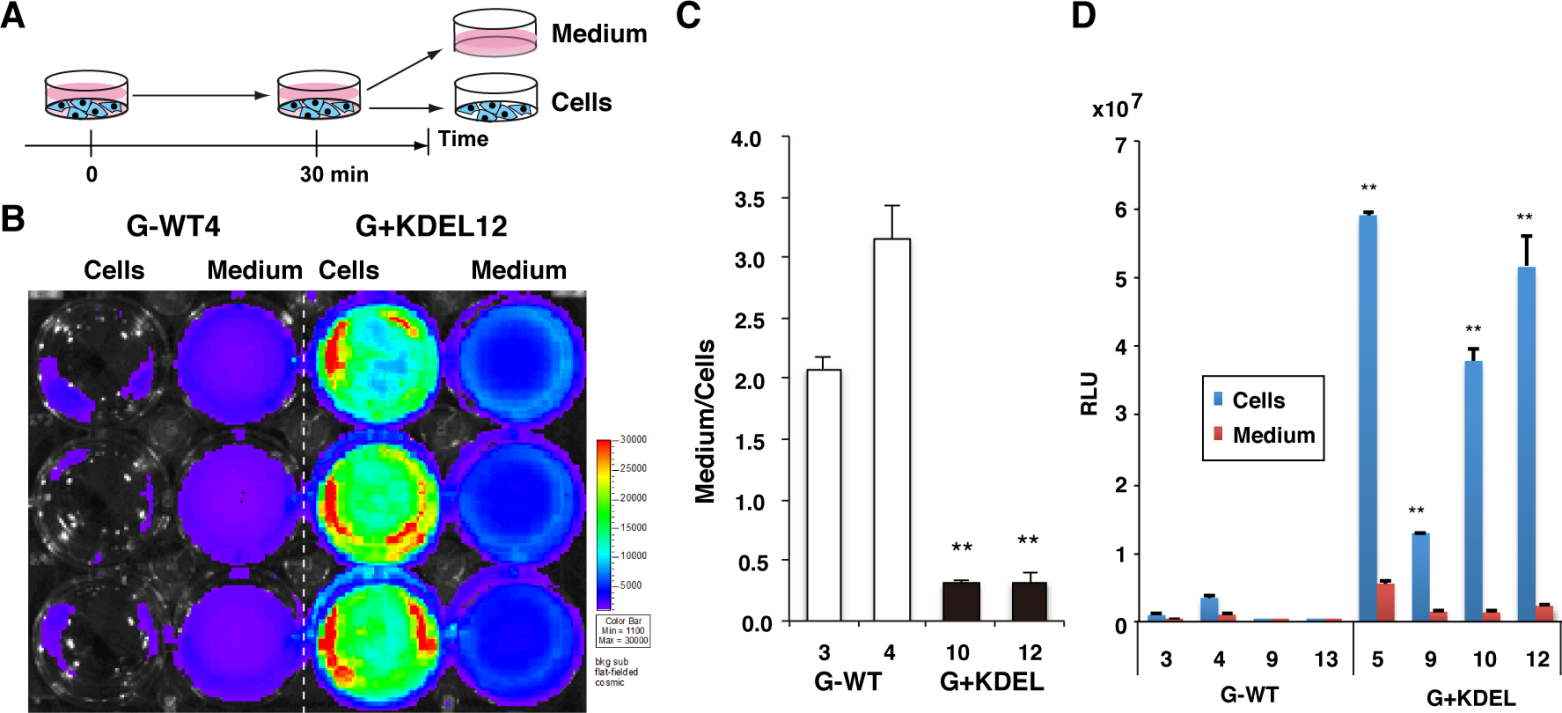
Strong intracellular bioluminescence in G-Luc+KDEL transgenic HepG2 cells. (A) Experimental scheme of the cytotoxicity test presented in this study. (B) IVIS luminescence images of transgenic HepG2 clones: G-WT4, a HepG2 cell clone expressing G-WT; G+KDEL12, a clone expressing G+KDEL. Cells exhibiting strong G-Luc activity (red or yellow) were observed only in the G+KDEL12 variant clone, reflecting the enzyme’s intracellular localization. (C) Ratio of luminometric activities of G-Luc in medium *vs*. cells, obtained using a microplate reader. Medium/Cells ratio was calculated after 48 hr culture. The significant reduction in the Medium/Cells ratio indicates that the transgenic clones expressed cytoplasmic G-Luc (n = 3 wells for each, **p < 0.001). (D) The G+KDEL variant produces > 30-fold higher bioluminescent signals than G-WT in transgenic HepG2 cells (n = 3 wells for each of four clones, **p < 0.001).

Next, we compared the properties of these transgenic cells. Luminescence in cells and medium was visualized and measured by IVIS (Fig 3A). A significant reduction in the Medium/Cells ratio was detected in G+KDEL12, reflecting the strong cytoplasmic localization of G+KDEL (n = 3 wells, triplicate measurements for each well; 0.38 ± 0.08 in G+KDEL clones; 3.16 ± 0.27 in G-WT clones; *p* < 0.001) (Fig 3B and 3C). This observation was confirmed in other transgenic clones (G-WT3 and G+KDEL10) (Fig 3C). Moreover, signal intensities were obviously higher in G+KDEL cells than in G-WT cells. Luminometric analyses of four randomly selected clones for G+KDEL and G-WT revealed that G+KDEL had stronger enzymatic activity in cells than G-WT (n = 3 wells for each of four clones, >30-fold change, *p* < 0.001) (Fig 3D). In our system, a transgene is introduced into an identical genomic region of the host genome of each clone by Cre-loxP mediated site-specific recombination to minimize interclonal variation caused by position effects and variations in copy number. Thus, the stronger luminometric activity observed in G+KDEL HepG2 cells is likely because G+KDEL proteins have higher enzymatic activity and/or enzyme stability than G-WT in each cell. If these results are confirmed biochemically, G+KDEL could have broad use as a general and stable cytoplasmic reporter.

### Detection of dose-dependent changes in the cytolytic effects of carbon tetrachloride using G+KDEL transgenic HepG2 cells

G+KDEL12 HepG2 or G-WT4 HepG2 cells (2 × 10^5^ cells per well) were seeded in three wells of a 24-well plate for each dose of carbon tetrachloride (CCl_4_) (0.003% to 0.2%). CCl_4_ was dissolved in 1% dimethyl sulfoxide (DMSO), which was used as the solvent for all chemicals in this study.

**Fig 4:**
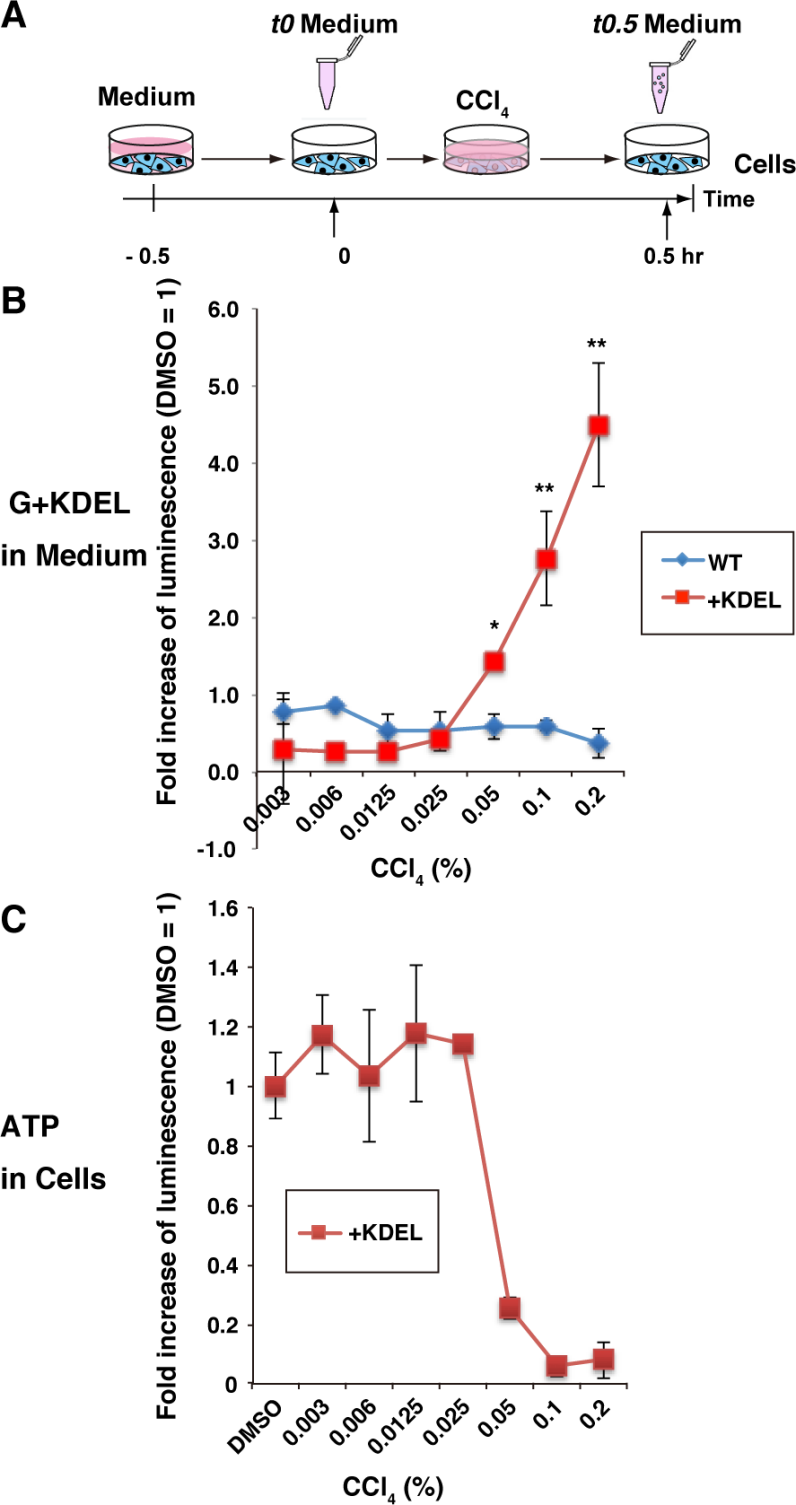
Dose-dependent effects of CCl_4_ on G+KDEL transgenic HepG2 cells. (A) Experimental scheme for analyzing dose-dependent effects of CCl_4_ on G+KDEL12 HepG2 cells. The WT4 HepG2 clone was used as a control. (B) Bioluminescence in the medium was detected using a microplate reader. Each RLU value was calculated relative to the value in the corresponding DMSO-treated control (defined as 1), and the means ± SD of three independent cultures were calculated (*p < 0.01; **p < 0.001). (C) Bioluminescence of “ATP in Cells” (CellTiter-Glo) detected using a microplate reader.

The culture medium was changed 0.5 hr before addition of CCl_4_, and then medium was collected as the 0 hr sample (*t0* Medium). Following addition of new medium containing CCl_4_ or 1% DMSO, medium was collected after 0.5 hr (*t0*.*5* Medium) (Fig 4A). To normalize for cell number variation between wells, the ratio of luminescent intensity in *t0*.*5* Medium *vs*. *t0* Medium was calculated. Moreover, values were further normalized against the value in the DMSO control, defined as 1. Normalized values greater than 1 indicated cytotoxicity of the chemical at the tested concentration. Relative to the WT control, G+KDEL12 cells exhibited a significant increase in luminescence upon treatment with CCl_4_ at concentrations from 0.05% (*p* < 0.01) to 0.2% (*p* < 0.001) (Fig 4B). By contrast, G-WT was not suitable for cytotoxicity test because no increase of luminometric intensity in the medium was detected following CCl_4_ treatment.

To confirm that extracellular G-Luc+KDEL fluorescence could accurately monitor cell death, we subjected the remaining cells to viability assays based on cellular ATP level (CellTiter-Glo, Promega). The two methods identified a range of minimum CCl_4_ concentration (0.025–0.05%) that result in cytotoxic effects under these conditions (Fig 4C).

### Detection of time-dependent changes in the cytolytic effects of carbon tetrachloride using G+KDEL transgenic HepG2 cells

**Fig 5:**
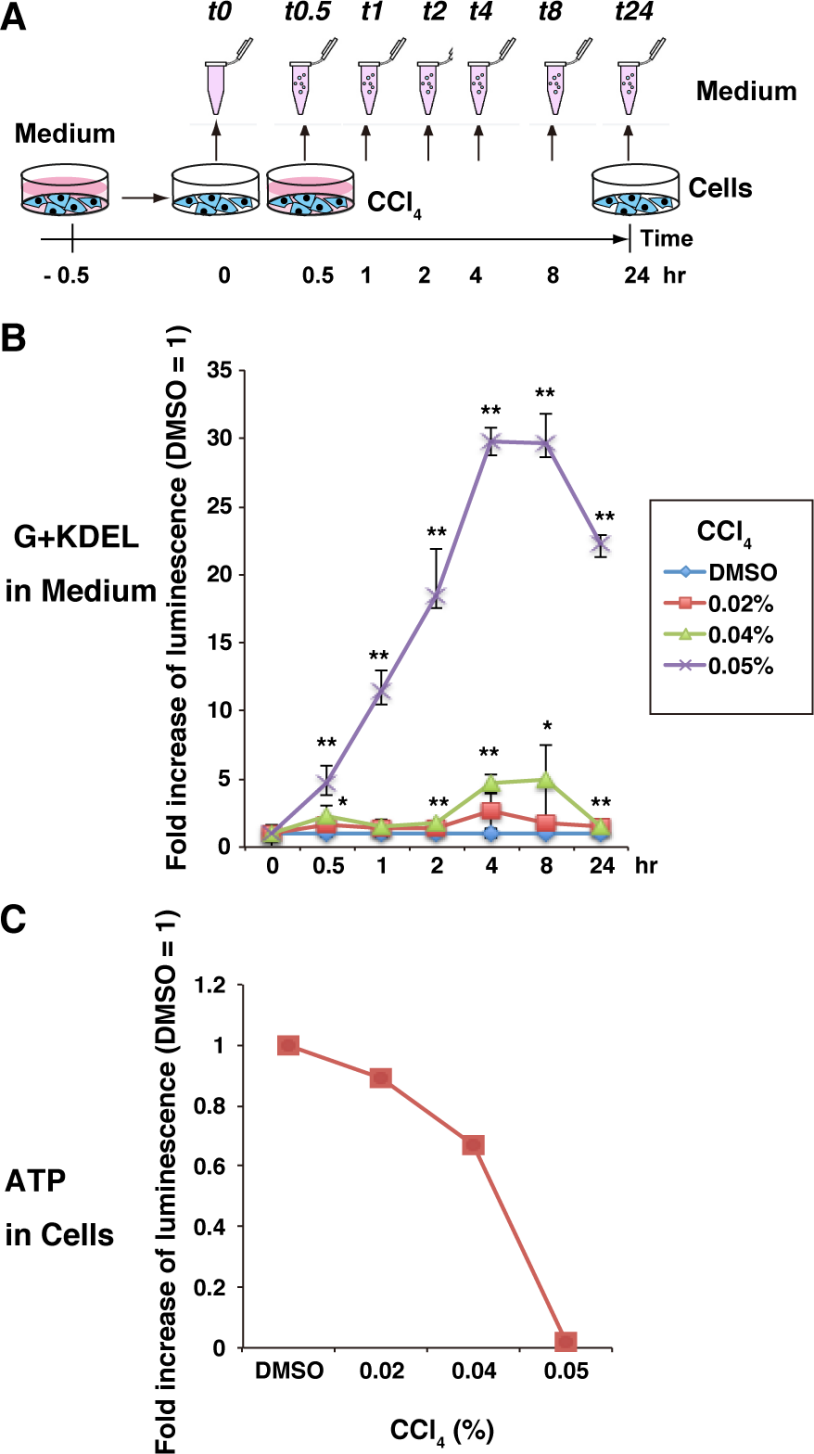
Time-dependent effects of CCl_4_ on G+KDEL transgenic HepG2 cells. (A) Experimental scheme for analyzing time-dependent effects of CCl_4_ on G+KDEL12 HepG2 cells. (B) Bioluminescence in the medium was detected using a microplate reader. Each RLU value was calculated relative to the value in the corresponding DMSO-treated control (defined as 1), and the means ± SD of three independent cultures were calculated (*p < 0.01; **p < 0.001). (C) Bioluminescence of “ATP in Cells” (CellTiter-Glo) was detected using a microplate reader, as shown in Fig 4C.

Next, we sequentially collected aliquots of medium 0.5 hr before treatment and at selected time points (0.5, 1, 2, 4, 8, and 24 hr) after treatment with 0.02%, 0.04%, or 0.05% CCl_4_ (n = 3) (Fig 5A). G+KDEL12 HepG2 cells treated with 0.05% CCl_4_ exhibited a significant increase in luminescence, reaching an intensity 30-fold greater than that of the DMSO-treated control 4–8 hr after the start of treatment (*p* < 0.001) (Fig 5B). In addition, relative to the DMSO-treated control, luminescence was also significantly increased after 2 hr of treatment with 0.04% CCl_4_ (*p* < 0.001) (Fig 5B). The CellTiter-Glo revealed that 0.02–0.05% CCl_4_ killed all of the cells by 24 hr (Fig 5C), whereas the G+KDEL system revealed that the cells were affected between 0.5–8 hr after the treatment (Fig 5B). However, when G+KDEL is used in endpoint assays (for example, at 24 hr after treatment), it may not be as advantageous as the CellTiter-Glo assay. The G+KDEL system is more appropriate for the analysis of the effects of acute exposure to cytotoxic chemicals or when time-dependent accumulation of cytotoxic effects needs to be estimated by repeated sampling at intervals of a few hours.

### Comparing the sensitivity of the G+KDEL system with those of other cytotoxicity assays

**Fig 6:**
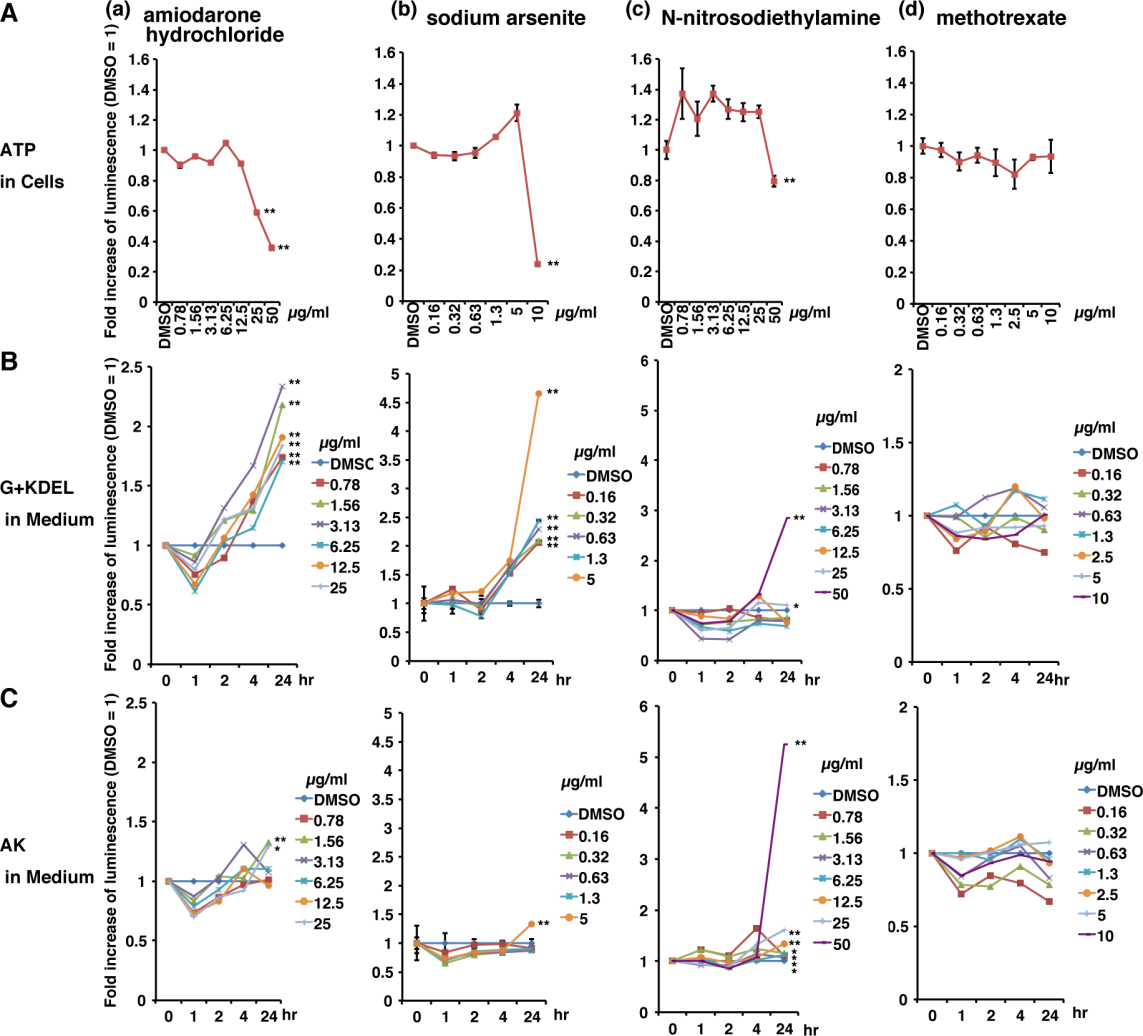
Comparison of the sensitivities of the G+KDEL-based assay and other tests to toxic drugs. (A) Cells were treated with (a) 0.78–50 μg/ml amiodarone hydrochloride; (b) 0.16–10 μg/ml sodium arsenite; (c) 0.78–50 μg/ml N-nitrosodiethylamine; or (d) 0.16–10 μg/ml methotrexate. Cells were subjected to the ATP assay (A). Medium was collected at five time points (0, 1, 2, 4, and 24 hr) and subjected to the G+KDEL assay (B) and AK test (C). Bioluminescence was detected using a microplate reader. Each RLU value was calculated relative to the value in the corresponding DMSO-treated control (defined as 1), and the means ± SD of three independent cultures were calculated. (n = 3 wells for each treatment; *, *p* < 0.01; **, *p* < 0.001).

In modern life, we are exposed to many chemicals, including therapeutic drugs, agrichemicals, and by-products from industrial processes. Therefore, we investigated whether G+KDEL12 HepG2 was sufficiently sensitive to detect the cytotoxicity of these chemicals even at low concentrations, and compared its sensitivity with those of other commercially available tests. We selected four drugs for which cytotoxicity data in humans were already available [21]. G+KDEL12 HepG2 cells (2 × 10^5^ cells/well) were cultured for 2 days in a 12-well plate, and then the medium was refreshed. After 1 hr, *t0* Medium was collected from every well of the plate. Next, the cells were cultured in medium containing various concentrations of drugs dissolved in 1% DMSO: 0.78–50 μg/ml amiodarone hydrochloride, an anti-arrhythmic drug (Fig 6(A)); 0.16–10 μg/ml sodium arsenite, an agrichemical (Fig 6(B)); 0.78–50 μg/ml N-nitrosodiethylamine, a carcinogenic industrial by-product (Fig 6(C)); and 0.16–10 μg/ml methotrexate, a drug used for chemotherapy of several cancers (Fig 6(D)). Aliquots of medium were collected 1, 2, 4, and 24 hr after the treatment (n = 3). All medium samples were divided in two groups and subjected to luminometric analyses.

Luminometric analyses of living cells were performed using CellTiter-Glo after 24 hr treatment, at the endpoint of this assay (Fig 6A(a)–(d)). The results of these assays identified minimum cytotoxic concentrations for three of the drugs: amiodarone hydrochloride, 25 μg/ml; sodium arsenite, 5 μg/ml; and N-nitrosodiethylamine, 25 μg/ml. Although methotrexate induces liver damage in rats *in vivo* [22], all three assay methods failed to detect any sign of cytotoxicity in the cells treated with this compound (Fig 6(D)).

Next, we precisely compared the sensitivities of the two types of cytotoxicity test based on levels of secreted enzymes, G+KDEL (Fig 6B(a)–(d)) and AK (Fig 6C(a)–(d)), in samples treated with the critical concentration for each chemical. The relative amount of AK was measured using the ToxiLight BioAssay Kit (Lonza).

In G+KDEL cells treated with amiodarone hydrochloride, G-Luc luminescence increased significantly after 4 hr. At 24 hr, cytotoxicity was observed in cells treated with >0.78 μg/ml amiodarone hydrochloride (n = 3, *p* < 0.001) (Fig 6A(a)), whereas the AK assay performed on identical samples revealed significant cytotoxicity only at 1.56 μg/ml (Fig 6C(a)). Similarly, the G+KDEL system was more sensitive than the AK system to toxicity induced by 4 hr treatment with 0.16–5 μg/ml sodium arsenite (n = 3, *p* < 0.001) (Fig 6(B)). By contrast, the AK assay had better sensitivity for the detection of cytotoxicity than the G+KDEL system; the AK assay detected cytotoxicity induced by 12.5–50 μg/ml N-nitrosodiethylamine, whereas the G+KDEL system detected cytotoxicity induced by 25–50 μg/ml (Fig 6(C)).

Thus, time-dependent accumulation of secreted G+KDEL12 from damaged cells increased the intensity of luminescence to detectable levels. The sensitivity and reliability of the G+KDEL12-based assay was comparable to, or indeed higher than, those of established tests.

## Discussion

Our system, using HepG2 cells expressing G+KDEL, provides a platform for easy and relatively sensitive cytotoxicity testing applicable to HCS. By contrast, HepG2 cells expressing C-Luc variants were not useful, because it was not possible to prevent secretion of C-Luc into the medium in the absence of cellular damage. In addition, elimination of the SP element from the N-terminus of this protein significantly decreased its luciferase activity. Elimination of SP also reduces the enzymatic activity of G-Luc [14]. Therefore, we concluded that the G+KDEL is the optimal cytoplasmic luciferase for use in cytotoxicity assays, because it is highly stable and has the highest luminometric activity among available luciferases.

The sensitivity of the G+KDEL-mediated cytotoxicity test described in this study could be greatly improved by increasing the expression level of the reporter gene. Expression level can vary depending on copy number in the transgenic HepG2 cells, position effects of the transgene integration site, and the strength of the promoter. In this study, we used the strong promoter CAG, resulting in high intracellular luminescence in transgenic HepG2 cells. We also tested a minimal mouse albumin promoter, but the resultant construct was much less sensitive to cytotoxic chemicals in transgenic HepG2 cells (data not shown). Therefore, transgenic HepG2 cells carrying several copies of pCAG-G+KDEL may provide more sensitive and accurate cytotoxicity tests, even at low concentrations of chemicals, in a shorter time and using fewer cells (e.g., in > 96-well format).

Transcriptional repression generally occurs in cells treated with cytotoxic reagents, mainly as a result of cell cycle arrest. This results in reductions in the levels of newly-synthesized mRNA, endogenous proteins, and/or CAG promoter-driven reporter proteins. Thus, transcriptional repression of G+KDEL at mRNA levels can be detected and used in cytotoxicity assays. However, in the secreted enzyme-based assay, almost all cells would already contain comparable amounts of cellular proteins and the assay is usually completed within 24 hrs. Cytotoxic agents, therefore, might have minimal effects on G+KDEL transcription in the enzyme-based assay.

HepG2 cells have been widely used as a model system for studies of direct and/or indirect cytotoxicity of xenobiotics, hepatocarcinogenesis, and drug targeting, and they have been extensively analyzed in regard to their abilities to activate or detoxify xenobiotics. The results of these studies showed that HepG2 cells are protected from many cytotoxic and DNA-damaging xenobiotics by upregulation of detoxifying enzymes, interactions with DNA-repair and/or replication processes, and induction of metabolic activities. Although the liver metabolic function of HepG2 cells is inferior to that of human hepatocytes, they reflect the *in vivo* metabolism of xenobiotics in humans more accurately than other metabolically incompetent cells used in *in vitro* testing, and may thus provide a clearer understanding of the properties of target reagents than conventionally used cells. Consequently, they are especially well suited for the detection of cytotoxic and genotoxic chemicals [7]. Therefore, we used HepG2 cells to test the properties of G-Luc+KDEL. We also used G+KDEL12 HepG2 cells to test acetaminophen (APAP), a pain and fever reducer that is converted into a toxic by-product, N-acetyl-p-benzoquinone imine (NAPQI), mainly by the P450 enzyme CYP2E1 [23]. However, we obtained no signal in the effective concentration range (data not shown). The APAP results indicate that HepG2 cells lack an important metabolic activity, and are therefore imperfect models of human hepatocytes. Therefore, a reliable HCS-based hepatotoxicity test can be established only by applying the G+KDEL12 reporter system to other cell lines with metabolic activities similar to those of human adult primary hepatocytes. Previously, we produced transgenic HepaRG cells that differentiate into adult hepatocyte-like cells and express a strong GFP-derived reporter under the control of the adult hepatocyte-specific promoter of CYP3A4 [19]. In the near future, improved matching of reporters and biomaterials should provide more accurate quantitative HCS-based cytotoxicity testing.

## Materials and Methods

### Vector construction

First we produced a basic plasmid vector, *i*.*e*., a shuttle vector, that contains the following major elements: a gene expression unit driven by the CAG promoter, placed between two INS; a neomycin-resistance gene driven by the human PGK promoter; and a loxP site. A multiple cloning site (MCS) including the *Bam*HI, *Bsr*GI, *Mlu*I, and *Mfe*I (*Mfe*I results in ends that are compatiblewith those of *Eco*RI) restriction sites, in that order, was inserted adjacent to the CAG promoter. The ORF of G-Luc, isolated from the pCMV-G-Luc2 control plasmid (New England Biolabs) by digestion with *Bam*HI and *Xba*I, was inserted between the *Bam*HI and *Mfe*I sites in the MCS by blunting the *Xba*I and *Mfe*I ends. The G+KDEL ORF was digested with *Nhe*I and *Eco*RI; the *Eco*RI site was derived from vector pGEM-T (Easy) (Promega) and inserted into MCS by blunting the *Nhe*I end. ORF fragments for the C-WT and the C-Luc variants, including the stop codon, were PCR amplified from the pCMV-C-Luc2 control plasmid (NEB) using a forward primer with a *Bam*HI-*Bsr*GI linker and a reverse primer with an *Mlu*I-*Mfe*I linker. Primer sets used in this study are summarized in Table 1.

### Generation of transgenic cells

Each of the five types of expression vector was introduced into the loxP site in loxP-HepG2 cells *via* Cre-loxP-mediated site-specific recombination. The loxP-HepG2 cells were previously created by a random insertion of a loxP-bearing plasmid vector into the q13-21 region of human chromosome 14 [19]. loxP-HepG2 cells were seeded on 35 mm culture dishes, and 2 μg of purified vector was co-transfected with 1 μg of the Cre expression vector pCAG-Cre using Lipofectamine® LTX (Life Technologies, Thermo Fisher Scientific). On the following day, the cells were replated in three 100 mm culture dishes. Transgenic clones, which were resistant to G418 due to the Pgk-*neo* gene in the shuttle vector, were selected by culture in 800 μg/ml G418-containing medium. More than 10 G418-resistant clones were picked for each of the five vectors, expanded, and CHARACTERIZED by genomic PCR and luminometric analyses. Genomic DNA was extracted from cultured cells using the Gentra Puregene Cell Kit (QIAGEN), and PCR was performed in 20 μl of TaKaRa Ex Taq mixture (TaKaRa Bio) on a GeneAmp PCR system 9700 (Applied Biosystems). Primer sets used for genomic PCR are summarized in Table 1.

### Cell culture and chemicals

HepG2 cells were seeded at a density of 1 × 10^5^ cells/cm^2^ on culture dishes in Dulbecco’s modified Eagle’s medium (Wako) supplemented with 10% (v/v) fetal bovine serum (Biowest), and then cultured in 5% CO_2_ at 37°C. Reagents were purchased from the indicated suppliers: ampicillin, DMSO, and amiodarone hydrochloride (Sigma-Aldrich); sodium arsenite (Merck-Millipore); G418 (Calbiochem); and MG132, CCl_4_, N-nitrosodiethylamine, APAP, and methotrexate (Wako). All reagents used in cytotoxicity tests were dissolved in 1% DMSO.

### IVIS *in vivo* imaging

For IVIS analyses, transgenic HepG2 cells were seeded at a density of 1 × 10^5^ cells/cm^2^ 1 day before use. For each clone, cells were seeded in at least three wells for triplicate measurements. Thirty minutes before the assay, the medium was completely replaced with fresh culture medium (400 μl per well for 24-well plates; 1 ml for 35 mm dishes). Within 30 min, culture medium containing secreted Luc was transferred to a new well and tested as “Medium”. At the same time, the same amount of fresh culture medium was again added to the cells, and the sample was immediately tested as “Cells”. Substrates supplied in the BioLux® *Gaussia* Luciferase Assay Kit (NEB) for assaying G-Luc activity or the BioLux *Cypridina* Luciferase Assay Kit (NEB) for assaying C-Luc activity were added to both the “Medium”- and “Cells”-containing wells at the same time. Substrate concentration was carefully controlled because it significantly influences luminescence intensity. In this study, 50 μl and 20 μl of substrates for G-Luc and C-Luc, respectively, were used per 3 ml of medium. Luminescence images of transgenic HepG2 cells and culture medium were acquired after 30 min using an IVIS® Spectrum *in vivo* system (Caliper, PerkinElmer).

### Quantitation of luminescence

For luminometric analyses using a plate reader, transgenic HepG2 cells were seeded in most cases at 2 × 10^5^ cells/cm^2^ 2 days before use. Because secreted G-Luc and C-Luc are very stable, each G-Luc-containing sample was collected at sequential time points and centrifuged at 1,000 rpm for 3 min; the resultant supernatant was stored at 4°C until the endpoint of the assay. Ten microliters of stored supernatant was transferred into each of three wells of a white 96-microwell plate for each sample (Nunc, Thermo Fisher Scientific). After addition of 10 μl of the substrate described before, luminescence (RLU) was measured using Infinite500 (TECAN), a filter-based multimode microplate reader.

### Sensitivity comparison of cytotoxicity tests

Medium samples collected from transgenic HepG2 cells before and after chemical treatment were evenly divided and subjected to analysis of G-Luc and AK activities. Cell samples were used for ATP assays at the endpoint. AK and ATP activities were measured in parallel using the ToxiLight™ Non-Destructive Cytotoxicity BioAssay Kit (Lonza) and CellTiter-Glo® Luminescent Cell Viability Assay (Promega), respectively.

### Statistical analyses

Because sample size was limited and it was not clear whether the data were normally distributed, the Mann-Whitney U-test was used to evaluate differences. For convenience, significance was determined using Z values, which were calculated using Microsoft Excel. When the absolute value of a calculated Z score was greater than the absolute value of Z (2.58) and Z (3.29), the difference was deemed to be significant at the level of *p* < 0.01 (*) and *p* < 0.001 (**), respectively.
